# Sudden hearing loss caused by labyrinthine hemorrhage

**DOI:** 10.1016/S1808-8694(15)31390-2

**Published:** 2015-10-17

**Authors:** Raquel Salomone, Taleb Abdu Ali Abu, Adriana Gonzaga Chaves, Maria Carmela Cundari Bocalini, Andy de Oliveira Vicente, Paulo Emmanuel Riskalla

**Affiliations:** 1 Medical resident in otorhinolaryngology, CEMA Hospital; 2 Medical resident in otorhinolaryngology; 3 Graduate student; 4 Otorhinolaryngologist, tutor in the medical residency program in otorhinolaryngology, CEMA Hospital and the Municipal Public Servant Hospital; 5 Master’s degree, doctoral student in otorhinolaryngology, UNIFESP/EPM. Tutor in the medical residency program in otorhinolaryngology, CEMA Hospital; 6 Master, adjunct professor in otorhinolaryngology, UNIFESP/EPM. Coordinator and tutor of the medical residency program in otorhinolaryngology. CEMA Hospital

**Keywords:** vertigo, hearing loss, sudden deafness

## Abstract

Sudden sensorineural hearing loss is relatively frequent. In most cases, the etiology is not discovered. One of the possible causes for sudden deafness is inner labyrinth bleeding, which was difficult to diagnose before the advent of magnetic resonance imaging. The purpose of this paper is to report a case of sudden hearing loss caused by a labyrinthine hemorrhage, and to present a review of the literature on this topic.

## INTRODUCTION

Sudden sensorineural hearing loss (SSHL) is generally unilateral, sudden, or rapidly progressive hearing loss,^1^ which affects about 15 thousand persons worldwide every year.^2^ The etiological diagnosis is usually polemic;^3^ about 85% of cases are catalogued as being of idiopathic cause, and are treated empirically. Spontaneous improvement of hearing loss occurs in 65% of patients within 2 weeks.^2^ Full recovery of hearing occurs in about 25% of patients; this is also the percentage of cases that do not improve.^1^, ^2^, ^3^ SSHL caused by intralabyrinthine hemorrhage is extremely rare, and there have been few such reports in the literature.

The aim of this paper was to report a case of SSHL probably caused by intralabyrinthine hemorrhage, in a Marfan’s syndrome patient that was under anticoagulation therapy. A review of the literature is also presented.

## CASE REPORT

E.F.G, a male patient aged 40 years, sought our unit and complained of sudden right-ear hearing loss that had started five days previously. There was not tinnitus, vertigo or aural fullness. Marfan’s syndrome had been diagnosed in this patient; he had undergone aortic valve heart surgery three years ago (metal valve). The patient had been prescribed a betablocker and regular oral anticoagulation medication.

**Figure 1:**
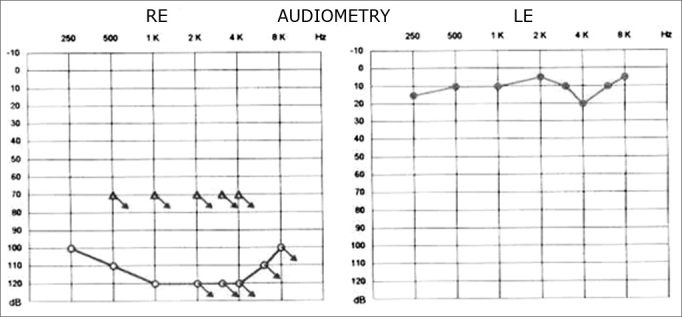
Pure tone audiometry before therapy.

The physical examination showed acromegaly; the heart auscultation evidenced the metal aortic prosthesis. The static and the dynamic balance were normal; there was no spontaneous or semi-spontaneous nystagmus. Pure tone audiometry revealed profound sensorineural hearing loss in the right ear (Figure 1). Coagulation tests on the day the patient was admitted into the hospital showed an INR of 4 and increased prothrombin time and activity. Axial and coronal magnetic resonance imaging slices of the temporal bones were obtained (Figures 3 and 4), which revealed a hyperintense cochlear signal in T1 (with no endovenous contrast), and a vestibule and the anterior portion of the lateral semicircular canal of the right ear typical of intralabyrinthine hemorrhage.

**Figure 2:**
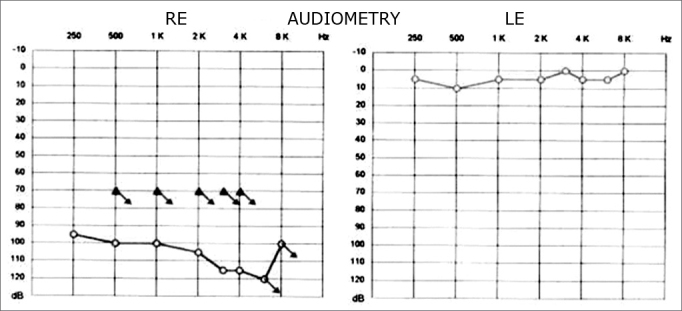
Pure tone audiometry after drug therapy.

**Figure 3:**
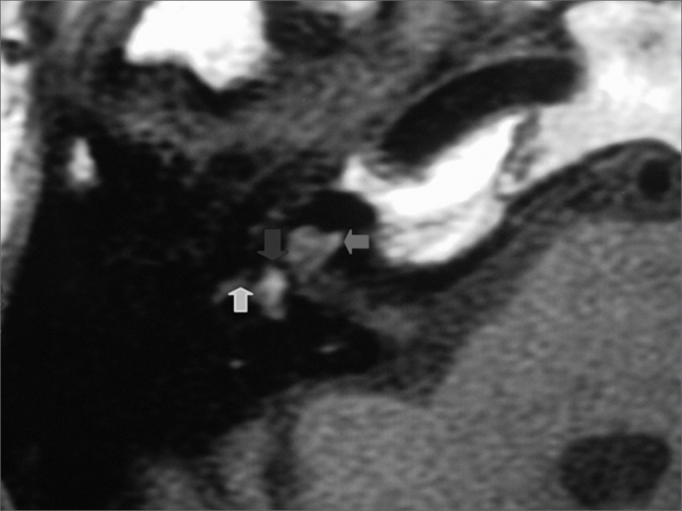
Magnetic resonance imaging of the temporal bones; axial slice, T1-weighted image with no endovenous contrast, showing a hyperintense signal in the cochlea (red arrow), the vestibule (blue arrow) and the anterior portion of the lateral semicircular canal (green arrow).

**Figure 4:**
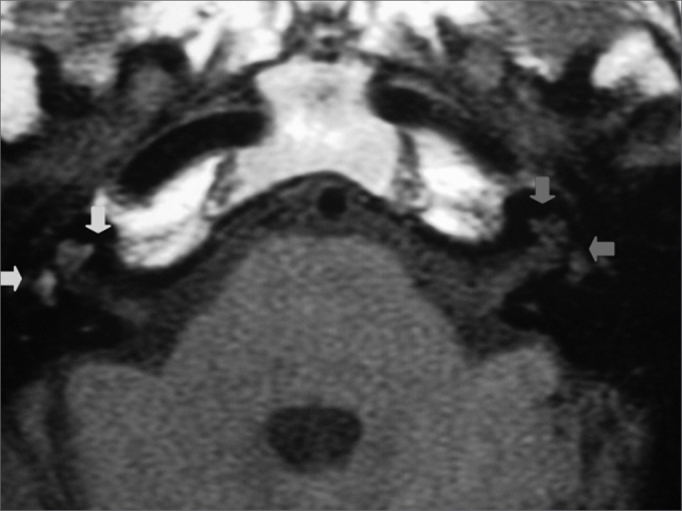
Magnetic resonance imaging of the temporal bones; axial slice, T1-weighted image with no endovenous contrast, showing cochleo-vestibular signal hyperintensity in the right ear (green arrows); the contralateral ear shows normal signal intensity (red arrows).

The patient was treated medically, resulting in improvement of hearing (20 dB) and decreased vertigo.

## DISCUSSION

SSHL is defined as sensorineural audiometric loss of 30dB or more at three consecutive auditory frequencies, arising within not more than 72 hours.^2^, ^3^ It is typically unilateral,^4^ and may be partial or complete, and sudden or rapidly progressive.^1^

Causes of SSHL may be infectious (viral or bacterial), vascular, immunomediated (within the inner ear or systemically), or resulting from neurological disease (migraine, multiple sclerosis), neoplasms or ototoxicity.^3^

Four theories based on histopathology have been developed to explain the pathophysiology of SSHL: a viral infection, an immunological origin, membrane rupture, and circulation disorders.^1^, ^2^, ^3^ The immunological theory postulates that inner ear cells are harmed by immunological complexes originating from systemic diseases. Some authors have suggested that viruses cause a direct cytotoxic effect on cochlear sensorial cells and/or induce the production of auto-immune complexes.^1^, ^2^, ^3^ A compromised blood supply to the inner ear sensorial cells may result in cochlear-labyrinthic ischemia, causing cell death within important inner ear structures.5 Rupture of Reissner’s membrane or the presence of perilymphatic fistulae may also cause SSHL.

The vascular etiology of SSHL may be hemorrhagic or obstructive. Intralabyrinthine hemorrhage (ILH) is a rare complication in patients that have hematological disease and/or under anti-coagulation therapy,^6^, ^7^ as described in this paper. The advent of magnetic resonance imaging made it possible to diagnose this condition with greater accuracy.

De Klein postulated that central vascular injury could be the cause of auditory disorders;^5^ Schucknecht et al. has stated that inner ear hemorrhage does not occur in healthy subjects.^8^

ILH has been described in patients with aplastic anemia,^9^ sickle-cell disease,^10^ leukemia,^9^, ^10^, ^11^ secondary to cranial trauma,^10^ and following surgery for the treatment of vestibular Schwannomas.^12^ Blood in the endolymph and the perilymph changes the hydrostatic pressure, which alters cochlear function and nerve stimulation.^13^ Kothari^6^ first described a case of ILH resulting from anticoagulation therapy.^6^ Our patient carried an artificial metal aortic valve, and required anticoagulation drugs.

T1-weighted MRI is valuable in the diagnosis of ILH. In normal subjects, the perilymph and the endolymph are isointense compared to the cerebrospinal fluid; the presence of fat, decreased blood flow, high protein concentration, or metahemoglobin^3^ generates hyperintense T1 images. ILH is more commonly found in the basal gyrus of the cochlea and next to the oval window.^14^ Fat-suppressed T1-weighted images may be used in differentiating ILH from lipomas (an uncommon condition).^3^, ^9^, ^14^

Until the current date, no study had been published attributing the cause of ILH to degeneration caused by Marfan’s syndrome.^15^

The treatment of SSHL remains extremely controversial even when the etiology is found. Clinical improvement attributed to hyperbaric oxygen therapy, anti-hypertensive drugs, anticoagulants, corticosteroids, plasma expanders, and hygienic-dietetic measures have not been shown to be superior to spontaneous cure.^1^, ^3^, ^14^, ^15^

## CONCLUSION

The abrupt onset of hearing loss associated with vertigo and the presence of a hypersignal in fat-suppressed T1-weighted MRI images of labyrinthic fluid strongly suggests acute intralabyrinthine hemorrhage. This minor hemorrhage may be the first complication of anticoagulation therapy.

In our patient, evidence suggests that sudden sensorineural hearing loss resulted from hemorrhage due to oral anticoagulation therapy, rather than possible morpho-physiological alterations typical of Marfan’s syndrome.
